# Assessing Weather Effects on Dengue Disease in Malaysia

**DOI:** 10.3390/ijerph10126319

**Published:** 2013-11-26

**Authors:** Yoon Ling Cheong, Katrin Burkart, Pedro J. Leitão, Tobia Lakes

**Affiliations:** 1 Geoinformation Science Lab, Department of Geography, Humboldt-Universität zu Berlin, Unter den Linden 6, Berlin 10099, Germany; E-Mail: tobia.lakes@geo.hu-berlin.de; 2 Medical Research Resource Centre, Institute for Medical Research, Jalan Pahang, Kuala Lumpur 50588, Malaysia; 3 Climatological Section, Department of Geography, Humboldt-Universität zu Berlin, Unter den Linden 6, Berlin 10099, Germany; E-Mail: katrin.burkart@geo.hu-berlin.de; 4 Geomatics Lab, Department of Geography, Humboldt-Universität zu Berlin, Unter den Linden 6, Berlin 10099, Germany; E-Mail: p.leitao@geo.hu-berlin.de

**Keywords:** dengue risk, weather effects, time-lag effects, generalized additive model (GAM), distributed non-linear lag model (DLNM), time series analysis

## Abstract

The number of dengue cases has been increasing on a global level in recent years, and particularly so in Malaysia, yet little is known about the effects of weather for identifying the short-term risk of dengue for the population. The aim of this paper is to estimate the weather effects on dengue disease accounting for non-linear temporal effects in Selangor, Kuala Lumpur and Putrajaya, Malaysia, from 2008 to 2010. We selected the weather parameters with a Poisson generalized additive model, and then assessed the effects of minimum temperature, bi-weekly accumulated rainfall and wind speed on dengue cases using a distributed non-linear lag model while adjusting for trend, day-of-week and week of the year. We found that the relative risk of dengue cases is positively associated with increased minimum temperature at a cumulative percentage change of 11.92% (95% CI: 4.41–32.19), from 25.4 °C to 26.5 °C, with the highest effect delayed by 51 days. Increasing bi-weekly accumulated rainfall had a positively strong effect on dengue cases at a cumulative percentage change of 21.45% (95% CI: 8.96, 51.37), from 215 mm to 302 mm, with the highest effect delayed by 26–28 days. The wind speed is negatively associated with dengue cases. The estimated lagged effects can be adapted in the dengue early warning system to assist in vector control and prevention plan.

## 1. Introduction

The risk of mosquito-borne dengue infection has increased dramatically in tropical and sub-tropical regions around the World in recent decades [1]. Each year there are between 50 and 100 million dengue infections, and more than 500,000 cases are hospitalized [2]. The pattern of dengue transmission is influenced by complex factors including the environment, climate and weather, human behavior and dengue virus serotype-specific herd immunity among the human population [3,4,5]. Here, we focus on weather, one of the fundamental driving forces behind dengue epidemics [6,7] that may allow us to narrow down the timeframe of high risk dengue infection. 

Dengue disease transmission is sensitive to weather for several reasons: a warm ambient temperature is critical to adult dengue vectors’ feeding behavior and gonotrophic cycle, as well as the rate of larval development and speed of virus replication; and rainfall-induced standing water are necessary for dengue vectors to breed [8,9,10]. The entire immature or aquatic cycle from egg to adult is approximately 7–9 days [11,12]. Dengue vectors become infected by biting infected humans or non-human primates (viremic stage), and they can then transmit the infection to other uninfected people after an extrinsic incubation period (EIP) of 8–12 days [13]. The EIP is the time when dengue vectors take a viremic blood meal to the time of the first successful transmission of the DENV [14]. After the intrinsic incubation period (IIP) of 4–10 days, the dengue symptoms begins unexpectedly sudden on the host [15]. The dengue vectors fit to transmit DENV survives for 30 days [16]. Hence, the estimated lagged time for the development of dengue vectors to the onset of dengue symptoms in human could be as short as 19 days if the dengue vectors bite a susceptive host on the first day after EIP. 

Many studies have reported varying associations and lagged effects between climate and weather on dengue cases. Strong positive correlations were found between El Niño-Southern Oscillation (ENSO) and dengue epidemics in 10 island nations of the South Pacific [17], across the Indonesian archipelago and northern South America [18], and in Thailand [19]. For temperature, a varying lagged effect was reported in countries situated within 13 and 25 degrees latitude, both North and South. An increasing dengue risk was associated with increasing minimum and maximum temperature by a 1–2 month lag in Mexico [20], Brazil [21] and French West Indies [22], but with a longer lag time up to 3–4 months in Barbados [23] and Australia [24]. For countries closer to the equator, that is, within 1–6 degrees North and South, a shorter 2-week lag of temperature on dengue cases was reported in Singapore [25], and a 1-month lag of temperature was reported in Indonesia [26]. Furthermore, rainfall interactions exhibited a mixture of influences, from a 2-week lag, a 4-week lag, a 7-week lag, to a 10-week lag on the increase of dengue cases in Mexico [27], Thailand [28], Barbados [23], and Taiwan [29], respectively. Wind speed also exhibited a disparate association with dengue cases, from the common negative association in Barbados [23], Sri Lanka [30] and Guangzhou, China [31], to no association in Thailand [32] and Taiwan [33]. This study contributes to the overall estimation of the weather influence on dengue transmission in the area close to the equator.

Earlier studies have shown that Malaysia is dengue hyperendemic, with all four serotypes circulating concurrently [34], and with an abundance of both *Aedes aegypti* and *Aedes albopictus* [35,36]. The potential contribution of the results in this work may help health workers and stakeholders to plan vector control activities. Few studies in Malaysia focus on the weather interaction with dengue vectors abundance [37,38,39,40] and dengue cases in a local study site [41,42]. To date, there is no study on the short-term weather interaction with dengue cases in Selangor, Kuala Lumpur and Putrajaya, Malaysia. We aimed to estimate the weather effects on dengue disease accounting for non-linear temporal effects. 

## 2. Materials and Methods

### 2.1. Study Area

**Figure 1:**
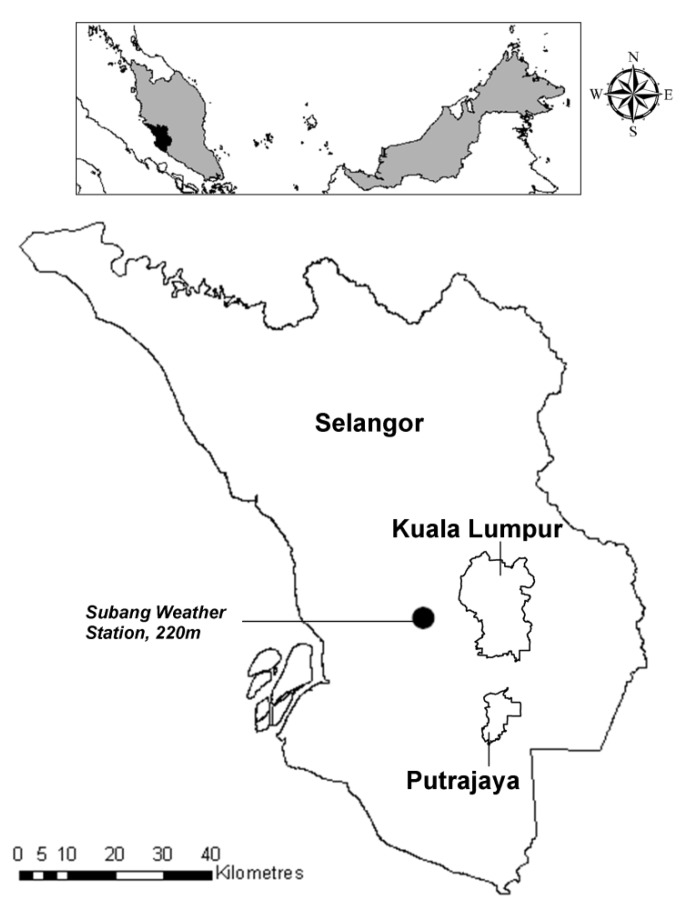
Study area: State of Selangor, including the federal territory of Kuala Lumpur and the federal administrative capital of Putrajaya.

The study area included the State of Selangor, the federal territory of Kuala Lumpur and the federal administrative capital of Putrajaya, and covered an area of 8,222 km^2^; the geographical location is between 2°35'N and 3°60'N, and 100°43'E and 102°5'E (Figure 1).

The tropical climate is characterized by fairly high but uniform average daily temperatures ranging from 21 °C to 32 °C, a mean annual temperature of 26 °C, average daily humidity levels exceeding 80%, and mean annual rainfall of about 2,500 mm. The climate of Selangor, Kuala Lumpur and Putrajaya is governed by two monsoonal winds, which originate from the northeast between October and February, and the southwest from May to September [43].

### 2.2. Data

In Malaysia, dengue is a nationally notifiable disease and physicians must report every suspected case of dengue to the local health authority within 24 h [44]. We obtained dengue data from the Disease Control Division, Ministry of Health Malaysia. We used only dengue cases that were confirmed by the serological tests IgM capture enzyme-linked immunosorbent assay (ELISA) with single positive IgM as also applied by other studies [45,46]. For Selangor, Kuala Lumpur and Putrajaya, 32,181 cases of dengue were found from 2008 to 2010. The number of dengue cases shows a mean of 29.4 per day, with a standard deviation of 13.7 (Table 1). 

**Table 1:** Distribution of dengue cases and selected weather parameters in Selangor, Kuala Lumpur and Putrajaya, 2008–2010.

Variables (unit)	Mean	Standard deviation	Minimum	Percentiles			
				25th	50th	75th	100th
Daily total dengue cases	29.4	13.7	1.0	20.0	28.0	38.0	117.0
Daily minimum temperature (°C)	24.2	1.0	20.4	23.5	24.0	24.9	27.0
Daily maximum temperature (°C)	32.9	1.6	25.4	32.0	33.0	34.0	36.4
Daily mean temperature (°C)	27.8	1.3	23.3	26.9	27.8	28.8	31.3
Daily relative humidity (%)	78.0	6.0	59.9	73.6	78.5	82.6	93.5
Cumulative bi-weekly rainfall (mm)	117.4	72.9	1.3	55.6	108.2	170.4	329.2
Daily mean wind speed (knots)	2.9	0.9	1.0	2.3	2.7	3.4	6.2

We compiled the daily data for maximum, minimum and mean temperature (in degrees Celsius), cumulative bi-weekly rainfall (mm), relative humidity (percentage) and mean wind speed (knots) from the local weather station in Kuala Lumpur, Subang (WMO# 486470; North Latitude 3°07'01''; East Longitude 101°32'60''; 220 masl) from 2007–2010. The data distribution of dengue cases and weather parameters are shown in Table 1. We used cumulative bi-weekly rainfall to include the immature cycle of dengue vectors that takes at least seven days, which is consistent with previous studies [47]. One station data was used due to the availability of the data, the high number of dengue cases concentrated near the station, and the weather conditions would not vary significantly across space [48]. Weather data were obtained from the National Climatic Data Center (NCDC) website [49]. 

### 2.3. Statistical Analysis

Initially, we included daily minimum temperature, daily maximum temperature, daily mean temperature, daily relative humidity, daily mean wind speed and bi-weekly rainfall in our analysis. We assessed the correlation analyses between all weather parameters and dengue cases. Mean temperature was reported with a high positive correlation with maximum temperature, and was then excluded (Table S4). 

We assessed the relationship between the weather parameters and the number of daily dengue cases using Poisson generalized additive models (GAM) [50] in the “mgcv” R [51] package, version 1.7–23 [52] with natural cubic splines. The GAM are useful for identifying non-linear relationships and do not require an a priori knowledge of the shape of the response curves [50,53], which is determined by the data itself [54]. We excluded outliers of 4 standard deviations from the mean for all weather parameters, as GAM modeling is outlier-sensitive [55]. Model construction was based on a stepwise forward and backward variable selection using the Akaike’s Information Criteria (AIC) score [56]. The significance of the spline term(s) was assessed and fitted with linear interactions when non-significance was detected. The best parsimonious model was selected based on the Delta AICs (AIC—minimum AIC) [56], and its accuracy was assessed by a 10-fold cross-validation. We assessed the autocorrelation and partial autocorrelation of the model residuals to adjust the need to account for seasonal trends. 

To capture the delayed effects of weather parameters on the number of dengue cases, we used distributed lag non-linear models (DLNM) in the “dlnm” R [51] package version 1.6.8 [57,58] to simultaneously describe non-linear and delayed dependencies in the association between weather parameters and dengue cases based on a “cross-basis” function. Recent studies have shown promising modeling performances in the weather effects on inpatient mortality and outpatient visit with GAM and DLNM [59,60,61,62]. We used lags up to 90 days to account for any potential lag period (*i.e.*, the extrinsic incubation period of the dengue vector and intrinsic incubation period of dengue virus). The median value of weather parameters (Table 1) was defined as the baseline centering value for calculating relative risk. The relative risk was based on the Poisson regression models adjusting for various confounders following the work of Gasparrini *et al*. [63]. We compared the relative risk at specific lags to account for the effect of the current day’s weather parameters on the current day’s dengue cases (lag 0), weather parameters one month before (lag 30), two months before (lag 60), and three months before (lag 90) on the current day’s dengue cases. To quantify the nonlinear exposure-response curves, we calculated the percentage change with the 95% confidence intervals (CIs) in the number of dengue cases for minimum temperature, cumulated rainfall and wind speed with the 99th percentile relative to the 90th percentile for high weather effect, and the 1st percentile relative to the 10th percentile, respectively, for low weather effect. The percentage change was calculated by the following formula (Equation 1) [64]:

Percentage Change = (Relative Risk − 1) × 100%(1)

Sensitivity analyses were performed by varying the degrees of freedom (df), using 3–7 df for trend adjustment. Moreover, we conducted the analysis for maximum lags of 60 and 90 days for the DLNM.

## 3. Results

### 3.1. Best Model Selection and Validation

By comparing the AIC and the Delta AIC values, we identified the best Poisson GAM model with minimum temperature, bi-weekly accumulated rainfall, and wind speed (AIC: 7367.23; deviance explained: 75.5%) (Tables S2 and S3). The relative humidity and maximum temperature were not statistically significant and were thus not included in the model (Table S4). The model was adjusted with a natural cubic spline of the time per year using 4 df, a factor for day of week and a natural cubic spline of week of the year to control for seasonal and long-term trends. We found that the selected GAM model correctly described 66% of the withheld deviance in a 10-fold cross-validation without a lag effect. The deviance not described by the model may account for temporal fluctuations in the immunity status of host populations [65], socio-economical factors [66], and other factors related to the population of dengue virus vectors [5]. The autocorrelation and partial autocorrelations of residuals from our main models were free from systematic patterns and summed close to zero (Figure S1). This suggested our original choice of smoothing had adequately adjusted for seasonal trends.

In the sensitivity analyses for DLNM, the estimates for the results with varying df and lag changed little. Hence, we eventually used the natural cubic B-spline with 3 df for minimum temperature, 3 df for accumulated rainfall, and 3 df for wind speed to describe the association of weather parameters and dengue cases. For the lag stratification, we selected 3 df for minimum temperature, 3 df for accumulated rainfall, and 3 df for wind speed. 

### 3.2. Association of Temperature and Dengue

**Figure 2:**
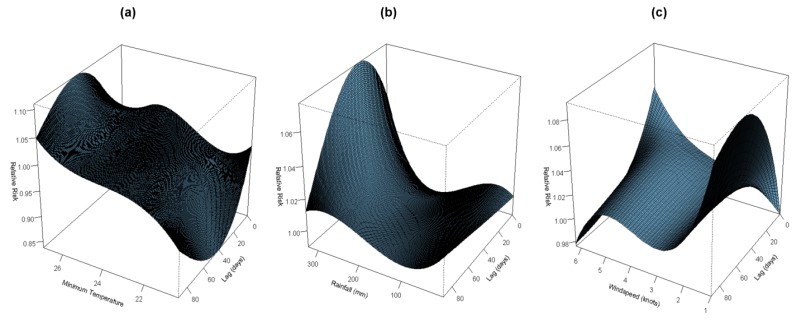
Relative Risk of dengue cases by (**a**) minimum temperature (°C); (**b**) rainfall (mm) and (**c**) wind speed (knots) for a lag of 90 days, using a “natural cubic B-spline-natural cubic spline” DLNM with a 3 degrees of freedom natural cubic B-spline for minimum temperature, a 3 degrees of freedom natural cubic B-spline for accumulated rainfall, and a 3 degrees of freedom natural cubic B-spline for wind speed. The reference values were median of minimum temperature (24 °C), rainfall (108.20 mm), and wind speed (2.7 knots). (see also Supplemental Movie S1, Movie S2 and Movie S3).

The estimated effects of minimum temperature were nonlinear for dengue cases, with increasing relative risk at a higher minimum temperature (Figure 2a). 

**Figure 3:**
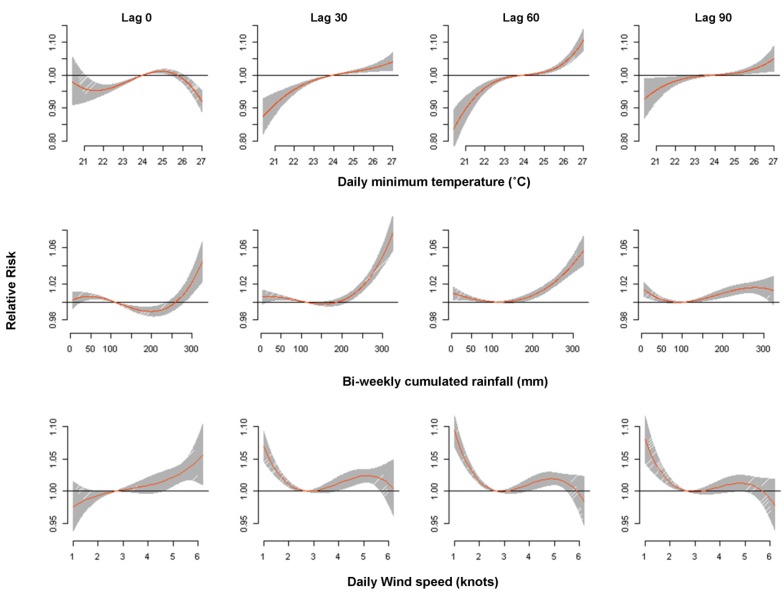
Relative Risk of dengue cases by minimum temperature (°C), rainfall (mm) and wind speed (knots) at specific lags of 0, 30, 60 and 90 days, using a “natural cubic B-spline-natural cubic spline” DLNM with a 3 degrees of freedom natural cubic B-spline for minimum temperature, a 3 degrees of freedom natural cubic B-spline for accumulated rainfall, and a 3 degrees of freedom natural cubic B-spline for wind speed. The reference values were median of minimum temperature (24 °C), rainfall (108.2 mm), and wind speed (2.7 knots).

The minimum temperature effect on dengue cases on the current day showed a different shape from the other specific lags of 30, 60 and 90 (Figure 3). The current day effect was basically not statistically significant, but the other lagged effect showed an increasing risk with an increase of minimum temperature. The increase of minimum temperature from 25.4 °C (90th percentile) to 26.5 °C (99th percentile) increased dengue cases by the highest amount, that is, 5.04% (95% CI: 3.58, 6.51) at a lag of 51 days (Table S4). The cumulative effect of the overall percentage change in the daily dengue cases exhibited a higher percentage value, of 11.92% (95% CI: 4.41–32.19) in warm temperature (an increase from 25.4 °C to 26.5 °C), than did the cold temperature (a decrease from 23 °C to 22 °C), at 0.10% (95% CI: 0.05–0.23) (Table S4). 

### 3.3. Association between Rainfall and Dengue

The estimated effect of rainfall on dengue cases obviously differed for low and high cumulated rainfall for a lag period of 90 days in the three-dimensional plot (Figure 2b). There was a strong effect at high rainfall, but a rather small effect at low rainfall (Figure 2b). Further, Figure 3 exhibited the strongest effect of rainfall at a lag of 30 days with a rapid increase of risk above bi-weekly cumulated rainfall of 200 mm. The increase of cumulated rainfall from 215 mm (90th percentile) to 302 mm (99th percentile) increased dengue cases by the highest, 4.75% (95% CI: 3.50, 6.01) at a lag of 26 to 28 days (Table S4). Overall, high rainfall exhibited a higher cumulative percentage value of 21.45% (95% CI: 8.96, 51.37) than the low rainfall level of 1.08% (95% CI: 0.94, 1.25) (Table S4). 

### 3.4. Association of Wind Speed and Dengue

The three-dimensional plot shows that the relative risk of dengue cases are inversely associated with the wind speed for longer lag periods (Figure 2c). The effect of low wind speed lasted for a longer period, while the effect of a high wind speed lasted for a shorter period (Figure 2c). For the high wind speed, the high effects were the largest at a lag of 0, and then declined gradually. Figure 3 exhibits the wind speed effect on dengue cases during the current day differed from the lagged effect. The relative risk of dengue cases increased with the increasing wind speed on the current day. At the lag of 1, 2 and 3 months, wind speed was negatively associated with dengue cases up to 3 knots, positively associated from 3–5 knots, followed by a negative association again at 5 knots and above. Table S4 shows the percentage change of wind speed on dengue cases. The drop of wind speed from 2.7 knots (10th percentile) to 1.7 knots (1st percentile) increased dengue cases by the highest amount, 4.02% (95% CI: 2.99, 5.06) at a lag of 59 days (Table S4). However, the increase of wind speed from 4.1 knots (90th percentile) to 5.7 knots (99th percentile) showed the highest effect at the lag of 0, 2.80% (95% CI: 0.12, 5.56), and then decreasing with the lagged period (Table S4). Overall, low wind speed exhibited a higher cumulative percentage value of 13.63% (95% CI: 5.42, 34.25) than the high wind speed of 1.30% (95% CI: 0.20, 8.39) (Table S4). 

## 4. Discussion

The aim of this study was to estimate the effects of weather parameters on dengue cases, with particular focus placed on lag times. Although the relationships between climate change effects on local weather and ecological systems is complex [67], it is encouraging that we found a short-term association of weather parameters, including minimum temperature, rainfall and wind speed with dengue cases at different lag periods.

We found the highest significant positive association between dengue cases and the minimum temperature with the lag time of 51 days, that is, close to two months. The significant association of dengue cases with minimum temperature was reported in numerous studies [21,23,68]. Similar observations were also reported in Taiwan [69] and Mexico [20], where minimum temperature at a lag of two months had the highest positive effect on dengue cases. The two-month lagged period may include time for dengue vectors to develop from eggs, become infected with the virus, EIP and biting activities in the gonotrophic cycle, and then IIP. Increasing temperatures shorten the gonotrophic cycle [5,70] and reduce the EIP [14,71,72]. At higher but not extreme temperatures, adult infected vectors require more blood meal to complete the gonotrophic cycle, and more than one gonotrophic cycle throughout the survival life cycle may lead to an increasing risk of dengue transmission [10,73]. Furthermore, a recent incubation period review stated that EIP decreases with increases in temperature [14]. In addition, a different pattern of risk observed for the current day and the lagged days might be due to the flying behavior of adult dengue vectors (Figure 3). A higher risk of dengue cases in colder temperatures exists, as dengue vectors tend to fly farther at 15 °C than at 27 °C, which leads to greater dispersal and a higher biting rate of humans [74]. 

Rainfall season is positively associated with DENV adult abundance and higher dengue transmission [5,75,76]. We found a higher risk of dengue cases reported during the lag of 26–28 days, or close to one month for bi-weekly cumulated rainfall (Table S4). This is in line with the studies that reported the highest risk of dengue cases related to rainfall 3 weeks prior in Veracruz, Mexico [77], and one month prior in Rio de Janeiro, Brazil [21], respectively. Rainfall influences the abundance of dengue vectors in the winged (adult) and aquatic populations (eggs, larvae, pupae) [7]. Increased near-surface humidity associated with rainfall enhances adult dengue vectors flight activity and host-seeking behavior [7], whereas increased rainfall supports more suitable breeding sites for the immature development of the aquatic population [78]. Furthermore, prolonged rainfall that leads to flooding may increase the dengue risk [79,80]. Aside from the indoor breeding habitats, probable rain filled breeding sites ranged from discarded car tires, animal watering dishes, tree holes, and discarded and neglected bottles and other containers are often found in parks, vacant land, blocked cement drains and septic tanks [78,81,82]. Moreover, the short lag period of one month indicated that the presence of a critical hyperendemic DENV environment in the study area may be due to the vertical dengue virus transmitting directly from adult to offspring [83], and multiple DENV serotypes co-circulating [34].

We found that wind speed is inversely associated with the dengue cases (Figure 2c, Figure 3), which is in line with the study in Barbados [23], Guangzhou, China [31] and Sri Lanka [30]. This was further supported by the higher cumulative percentage change in low wind speed compared to high wind speed (Table S4). Wind suppresses dengue vectors host-seeking flying activity, which affects oviposition and contact with humans [84,85,86]. However, a slight increase of dengue risk was observed with an increase in wind speed from 3 knots to 5 knots, as this is still within the maximum threshold where dengue vectors can fly freely, which was reported to have a threshold of 4.4 knots in Wisconsin [87]. The suitable wind condition below the maximum threshold favors the dispersion of dengue vectors and their oviposition [88]. Furthermore, there was a steep decrease of wind speed’s effect on dengue cases for a short period of 10–15 lag days at high wind speeds (Figure 2c). This pattern suggests some harvesting phenomenon, as also reported by other studies [57,89]. Therefore, short lags cannot adequately be used to assess risk effects [90]. A maximum percentage increase from 1.7 knots to 2.7 knots was found at the lag of 59 days. This lag period of 59 days was reasonable if we include the period for immature development, EIP, DENV infection and transmission, and IIP. 

Findings from our study can be adapted together with the other factors including population density, dengue virus circulating, efforts of vector control and vector density to assist in establishing a dengue early warning system. Current dengue disease control and prevention in Malaysia mainly based on the reported dengue cases in the passive surveillance system without predictive capabilities. For every notified dengue cases, adulticiding with space spraying is conducted. Other vector control strategies including larviciding with direct application of Temephos and misting of Bti [91], community-based larval control (COMBI) [92] and biological control [82] are applied for the selected outbreak areas or in areas where the outbreak cannot be controlled after long time period. In order to better control the dengue outbreak, an early warning system helps to alert on the increasing predicted risk of dengue based on the weather forecast and the other parameters to effectively target limited resources to the hotspot area [93]. This requires serious concern from local authorities, health professionals and the community to combine efforts for vector control and prevention. 

However, our study also has some limitations. Firstly, there was under-reporting of dengue cases to an unknown degree [94]. Even if there were unreported dengue cases, our study still provides insights, the pattern of dengue occurrences were consistent over time when we compared with the clinically suspected dengue cases (results not shown) and this is the only national surveillance data that is available. Secondly, due to data limitations we could not include unmeasured confounders such as variation in virus serotype and variation in dengue vectors population density. Nevertheless, in hyperendemic areas, analytical models based on syndromic case surveillance can be more informative than the vector densities, as the dengue virus transmission can occur even when dengue vectors population densities are low because of the repeating feeding behavior [72,95]. Thirdly, we used the aggregated number of dengue cases and weather parameters from one principal weather station. Using weather parameters as close to the highly clustered dengue cases area could reduce the spatial discrepancies between the hotspot area and the location of weather station. 

## 5. Conclusions

Temperature, rainfall and wind speed all influence dengue transmission in high population density areas. The estimated lagged effects and patterns, in accordance with the time necessary for the development of the dengue vectors, the EIP, and the incubation period in human body, as well as the onset of dengue symptoms, can be adapted in the vector control and prevention plan. The relationship found in this study helps to shed light on the link between weather and dengue for the development of future dengue prediction models while vaccines are not available. 

## Supplementary Files

Supplementary File 1 supplementary file 1 (PDF, 156 KB)

Supplementary File 2 supplementary file 2 (GIF, 4560 KB)

Supplementary File 3 supplementary file 3 (GIF, 5039 KB)

Supplementary File 4 supplementary file 4 (GIF, 5995 KB)
