# Supplementary material for: Assessing Weather Effects on Dengue Disease in Malaysia
Source: Int J Environ Res Public Health. 2013 Nov 26;10(12):6319–34. doi: 10.3390/ijerph10126319 (PMC3881116; doi:10.3390/ijerph10126319)
Supplement: Supplementary File 1 — (PDF, 156 KB) [file ijerph-10-06319-s001.pdf]

*Supplemental Information*

# Assessing Weather Effects on Dengue Disease in Malaysia

**Movie S1.** Relative Risk of dengue cases by minimum temperature (°C) for a lag of 90 days, using a "natural cubic B-spline-natural cubic spline" DLNM with a 3 degrees of freedom natural cubic B-spline. The reference median value was 24 °C.

**Movie S2.** Relative Risk of dengue cases by bi-weekly accumulated rainfall (mm) for a lag of 90 days, using a "natural cubic B-spline-natural cubic spline" DLNM with a 3 degrees of freedom natural cubic B-spline. The reference median value was 108.20 mm.

**Movie S3.** Relative Risk of dengue cases by wind speed (knots) for a lag of 90 days, using a "natural cubic B-spline-natural cubic spline" DLNM with a 3 degrees of freedom natural cubic B-spline. The reference median value was 2.7 knots.

We used 3D visualization device system (OpenGL rgl) in the "rgl" R [1] package version 0.93.986 [2] and software ImageMagick 6.8.7 Q16 to convert the result of Figure 2 to the animated movie.

**Table S1.** Pearson correlation coefficients between dengue cases and weathers parameters, Selangor, Kuala Lumpur and Putrajaya 2008–2010.

| | **Daily Minimum Temperature (°C)** | **Daily Maximum Temperature (°C)** | **Daily Mean Temperature (°C)** | **Daily Relative Humidity (%)** | **Daily Wind Speed (Knot)** | **Cumulative Bi-Weekly Rainfall (mm)** | **Daily Total Dengue Cases** |
|---|---|---|---|---|---|---|---|
| Daily minimum temperature | 1.00 | 0.47 | 0.64 | −0.09 | 0.11 | −0.11 | −0.29 |
| Daily maximum temperature | | 1.00 | 0.79 | −0.44 | 0.15 | −0.11 | −0.14 |
| Daily mean temperature | | | 1.00 | −0.60 | 0.28 | −0.18 | −0.22 |
| Daily relative humidity | | | | 1.00 | −0.38 | 0.33 | −0.09 |
| Daily wind speed | | | | | 1.00 | −0.19 | −0.09 |
| Cumulative bi-weekly rainfall | | | | | | 1.00 | −0.01 |
| Daily total dengue cases | | | | | | | 1.00 |

**Table S2.** Diagnostics of dengue-weather parameters models.

| Variable in the Model | AIC | Deviance Explained | Remarks |
| --- | --- | --- | --- |
| MinT | 7418.645 | 74.4 | All parameters statistically significant |
| MaxT | 7435.730 | 74.1 | All parameters statistically significant |
| WDSP | 7423.545 | 74.3 | All parameters statistically significant |
| RH | 7427.296 | 74.3 | All parameters statistically significant |
| RF | 7398.850 | 74.6 | All parameters statistically significant |
| RF, MinT | 7382.249 | 75.0 | All parameters statistically significant |
| RF, MaxT | 7396.225 | 74.7 | MaxT is not statistically significant |
| RF, RH | 7393.082 | 74.9 | All parameters statistically significant |
| RF, WDSP | 7383.954 | 75.0 | All parameters statistically significant |
| RF, MinT, MaxT | 7380.956 | 75.2 | MaxT is not statistically significant |
| RF, MinT, RH | 7378.860 | 75.3 | RH is not statistically significant |
| RF, MinT, WDSP | 7367.231 | 75.5 | All parameters statistically significant |
| RF, MinT, WDSP, RH | 7365.200 | 75.7 | RH is not statistically significant |
| RF, MinT, WDSP, MaxT | 7365.544 | 75.6 | MaxT is not statistically significant |

Models also include a natural cubic spline of time per year using 4 df, a factor of day of the week and a natural cubic spline of week of the year.

MinT = Minimum temperature; MaxT = Maximum temperature; WDSP = Wind speed; RH = Relative humidity; RF = Cumulative bi-weekly rainfall.

**Table S3.** Model selection based on delta AIC.

| Model | AIC | Delta AIC | Model Likelihood | Akaike Weight |
|---|---|---|---|---|
| minimum AIC model: RF, MinT, WDSP | 7367.231 | 0.000 | 1.000 | 0.996 |
| RF, MinT, RH | 7378.860 | 11.629 | 0.003 | 0.003 |
| RF, MinT, MaxT | 7380.956 | 13.725 | 0.001 | 0.001 |

MinT = Minimum temperature; MaxT = Maximum temperature; WDSP = Wind speed; RH = Relative humidity; RF = Cumulative bi-weekly rainfall.

The delta AIC ($\Delta_i$) is a measure of each model relative to the best model (Equation (1)).

$$\text{Delta AIC} = \Delta_i = AIC_i - \min AIC \quad (1)$$

where AIC$i$ is the AIC value for model $i$ , and min AIC is the AIC value of the best model that is with lowest AIC value. As a guideline, a $\Delta i < 2$ suggests substantial evidence for the model, values between 3 and 7 indicate that the model has considerably less support, whereas a $\Delta i > 10$ indicates that the model is very unlikely [3].

The delta AIC is 11.629, suggesting the model with $AIC_i$ is very unlikely to be selected as the best model. Hence, the best model (minimum temperature, rainfall and wind speed) is selected as the Akaike weight is 0.996.

**Table S4.** All percentage change (and 95% CIs) in the number of dengue cases for minimum temperature, cumulated rainfall and wind speed, with 99th percentile relative to 90th percentile and with 1st percentile relative to 10th percentile, respectively.

| Lag (Days) | Overall Percentage Change (95% CI) | | | | | |
|---|---|---|---|---|---|---|
| | Minimum Temperature (°C) | | Cumulated Rainfall (mm) | | Wind Speed (knots) | |
| | 1st Percentile (22 °C) Relative to 10th Percentile (23 °C) | 99th Percentile (26.5 °C) Relative to 90th Percentile (25.4 °C) | 1st Percentile (11 mm) Relative to 10th Percentile (24 mm) | 99th Percentile (302 mm) Relative to 90th Percentile (215 mm) | 1st Percentile (1.7 kn) Relative to 10th Percentile (2.7 kn) | 99th Percentile (5.7 kn) Relative to 90th Percentile (4.1 kn) |
| **Cumulative** | 0.10(0.05,0.23) | 11.92(4.41,32.19) | 1.08(0.94,1.25) | 21.45(8.96,51.37) | 13.63(5.42,34.25) | 1.30(0.20,8.39) |
| 0 | −2.00(−3.21, −0.78) | −4.69(−6.31, −3.05) | −0.19(−0.43,0.05) | 3.39(1.93,4.86) | −0.98(−2.74,0.81) | 2.80(0.12,5.56) |
| 1 | −2.06(−3.23, −0.89) | −4.30(−5.86, −2.72) | −0.18(−0.41,0.05) | 3.48(2.06,4.93) | −0.79(−2.49,0.93) | 2.70(0.09,5.38) |
| 2 | −2.12(−3.25, −0.98) | −3.91(−5.42, −2.39) | −0.18(−0.40,0.05) | 3.58(2.18,4.99) | −0.61(−2.26,1.06) | 2.61(0.06,5.22) |
| 3 | −2.18(−3.27, −1.08) | −3.53(−4.98, −2.05) | −0.17(−0.39,0.05) | 3.67(2.30,5.05) | −0.43(−2.02,1.19) | 2.51(0.02,5.06) |
| 4 | −2.24(−3.30, −1.16) | −3.14(−4.55, −1.72) | −0.17(−0.38,0.05) | 3.76(2.42,5.12) | −0.25(−1.79,1.32) | 2.41(−0.03,4.91) |
| 5 | −2.29(−3.33, −1.25) | −2.77(−4.13, −1.38) | −0.16(−0.37,0.05) | 3.85(2.53,5.18) | −0.07(−1.56,1.44) | 2.32(−0.09,4.78) |
| 6 | −2.35(−3.36, −1.32) | −2.40(−3.73, −1.05) | −0.16(−0.36,0.05) | 3.93(2.63,5.25) | 0.10(−1.34,1.57) | 2.22(−0.15,4.65) |
| 7 | −2.40(−3.40, −1.39) | −2.04(−3.34, −0.73) | −0.15(−0.35,0.05) | 4.01(2.73,5.31) | 0.27(−1.13,1.69) | 2.13(−0.22,4.53) |
| 8 | −2.45(−3.43, −1.45) | −1.69(−2.96, −0.40) | −0.14(−0.34,0.05) | 4.09(2.82,5.37) | 0.44(−0.93,1.82) | 2.04(−0.29,4.43) |
| 9 | −2.49(−3.47, −1.51) | −1.35(−2.60, −0.08) | −0.14(−0.33,0.05) | 4.16(2.90,5.43) | 0.60(−0.73,1.94) | 1.95(−0.37,4.33) |
| 10 | −2.54(−3.51, −1.56) | −1.02(−2.26,0.23) | −0.13(−0.32,0.06) | 4.22(2.97,5.49) | 0.75(−0.54,2.06) | 1.87(−0.45,4.24) |
| 11 | −2.58(−3.55, −1.60) | −0.70(−1.93,0.54) | −0.13(−0.31,0.06) | 4.29(3.05,5.54) | 0.90(−0.36,2.18) | 1.79(−0.53,4.16) |
| 12 | −2.62(−3.59, −1.64) | −0.39(−1.61,0.85) | −0.12(−0.31,0.06) | 4.34(3.11,5.59) | 1.05(−0.18,2.30) | 1.70(−0.62,4.08) |
| 13 | −2.66(−3.63, −1.68) | −0.09(−1.31,1.15) | −0.12(−0.30,0.07) | 4.40(3.17,5.64) | 1.20(−0.01,2.42) | 1.63(−0.70,4.01) |
| 14 | −2.69(−3.67, −1.71) | 0.20(−1.02,1.44) | −0.11(−0.29,0.07) | 4.45(3.22,5.69) | 1.33(0.15,2.53) | 1.55(−0.79,3.94) |
| 15 | −2.73(−3.71, −1.73) | 0.48(−0.75,1.73) | −0.10(−0.29,0.08) | 4.49(3.27,5.73) | 1.47(0.31,2.64) | 1.47(−0.88,3.88) |
| 16 | −2.76(−3.75, −1.76) | 0.76(−0.48,2.01) | −0.10(−0.28,0.09) | 4.54(3.31,5.77) | 1.60(0.46,2.76) | 1.40(−0.97,3.83) |
| 17 | −2.79(−3.79, −1.78) | 1.02(−0.23,2.29) | −0.09(−0.28,0.09) | 4.57(3.35,5.81) | 1.73(0.60,2.87) | 1.33(−1.06,3.77) |
| 18 | −2.81(−3.82, −1.80) | 1.28(0.01,2.56) | −0.09(−0.27,0.10) | 4.61(3.38,5.85) | 1.85(0.74,2.98) | 1.26(−1.15,3.72) |

**Table S4.** *Cont.*

| Lag (Days) | Overall Percentage Change (95% CI) | | | | | |
|---|---|---|---|---|---|---|
| | Minimum Temperature (°C) | | Cumulated Rainfall (mm) | | Wind Speed (knots) | |
| | 1st Percentile (22 °C) Relative to 10th Percentile (23 °C) | 99th Percentile (26.5 °C) Relative to 90th Percentile (25.4 °C) | 1st Percentile (11 mm) Relative to 10th Percentile (24 mm) | 99th Percentile (302 mm) Relative to 90th Percentile (215 mm) | 1st Percentile (1.7 kn) Relative to 10th Percentile (2.7 kn) | 99th Percentile (5.7 kn) Relative to 90th Percentile (4.1 kn) |
| 19 | −2.84(−3.86, −1.81) | 1.52(0.24,2.82) | −0.08(−0.27,0.11) | 4.64(3.41,5.88) | 1.97(0.87,3.08) | 1.19(−1.24,3.67) |
| 20 | −2.86(−3.89, −1.82) | 1.76(0.46,3.07) | −0.07(−0.26,0.11) | 4.66(3.43,5.91) | 2.08(0.99,3.19) | 1.12(−1.33,3.63) |
| 21 | −2.89(−3.92, −1.83) | 1.98(0.67,3.31) | −0.07(−0.26,0.12) | 4.69(3.45,5.93) | 2.19(1.11,3.29) | 1.05(−1.41,3.59) |
| 22 | −2.90(−3.96, −1.84) | 2.20(0.87,3.55) | −0.06(−0.25,0.13) | 4.71(3.47,5.95) | 2.30(1.23,3.39) | 0.99(−1.50,3.54) |
| 23 | −2.92(−3.98, −1.85) | 2.41(1.06,3.77) | −0.06(−0.25,0.13) | 4.72(3.48,5.97) | 2.41(1.34,3.48) | 0.93(−1.58,3.50) |
| 24 | −2.94(−4.01, −1.85) | 2.61(1.25,3.99) | −0.05(−0.24,0.14) | 4.73(3.49,5.99) | 2.50(1.44,3.58) | 0.87(−1.66,3.46) |
| 25 | −2.95(−4.04, −1.86) | 2.80(1.42,4.20) | −0.04(−0.24,0.15) | 4.74(3.50,6.00) | 2.60(1.54,3.67) | 0.81(−1.73,3.42) |
| 26 | −2.97(−4.06, −1.86) | 2.98(1.59,4.39) | −0.04(−0.23,0.15) | 4.75(3.50,6.01) | 2.69(1.64,3.76) | 0.75(−1.81,3.38) |
| 27 | −2.98(−4.08, −1.86) | 3.16(1.75,4.58) | −0.03(−0.23,0.16) | 4.75(3.50,6.01) | 2.78(1.73,3.84) | 0.70(−1.88,3.34) |
| 28 | −2.98(−4.09, −1.86) | 3.32(1.90,4.76) | −0.03(−0.22,0.17) | 4.75(3.50,6.01) | 2.87(1.82,3.93) | 0.64(−1.95,3.30) |
| 29 | −2.99(−4.11, −1.86) | 3.48(2.04,4.93) | −0.02(−0.22,0.18) | 4.74(3.49,6.01) | 2.95(1.90,4.01) | 0.59(−2.01,3.26) |
| 30 | −3.00(−4.12, −1.86) | 3.63(2.18,5.10) | −0.01(−0.21,0.18) | 4.73(3.48,6.00) | 3.03(1.98,4.08) | 0.54(−2.07,3.22) |
| 31 | −3.00(−4.13, −1.86) | 3.77(2.31,5.25) | −0.01(−0.21,0.19) | 4.72(3.47,5.99) | 3.10(2.06,4.16) | 0.49(−2.13,3.19) |
| 32 | −3.00(−4.14, −1.85) | 3.90(2.43,5.39) | 0.00(−0.20,0.20) | 4.70(3.45,5.97) | 3.17(2.13,4.23) | 0.44(−2.19,3.15) |
| 33 | −3.00(−4.15, −1.85) | 4.02(2.55,5.52) | 0.00(−0.19,0.20) | 4.69(3.43,5.95) | 3.24(2.20,4.29) | 0.40(−2.24,3.11) |
| 34 | −3.00(−4.15, −1.84) | 4.14(2.65,5.65) | 0.01(−0.19,0.21) | 4.67(3.41,5.93) | 3.30(2.26,4.36) | 0.35(−2.29,3.07) |
| 35 | −3.00(−4.15, −1.84) | 4.25(2.76,5.76) | 0.02(−0.18,0.22) | 4.64(3.39,5.91) | 3.37(2.32,4.42) | 0.31(−2.34,3.02) |
| 36 | −3.00(−4.15, −1.83) | 4.35(2.85,5.87) | 0.02(−0.18,0.22) | 4.61(3.37,5.88) | 3.42(2.38,4.48) | 0.26(−2.39,2.98) |
| 37 | −2.99(−4.15, −1.83) | 4.44(2.94,5.97) | 0.03(−0.17,0.23) | 4.58(3.34,5.85) | 3.48(2.44,4.53) | 0.22(−2.43,2.94) |
| 38 | −2.99(−4.14, −1.82) | 4.53(3.02,6.06) | 0.04(−0.16,0.24) | 4.55(3.31,5.81) | 3.53(2.49,4.58) | 0.18(−2.47,2.90) |
| 39 | −2.98(−4.13, −1.81) | 4.61(3.10,6.14) | 0.04(−0.16,0.24) | 4.52(3.28,5.77) | 3.58(2.54,4.63) | 0.14(−2.50,2.86) |

**Table S4.** *Cont.*

| Lag (Days) | Overall Percentage Change (95% CI) | | | | | |
|---|---|---|---|---|---|---|
| | Minimum Temperature (°C) | | Cumulated Rainfall (mm) | | Wind Speed (knots) | |
| | 1st Percentile (22 °C) Relative to 10th Percentile (23 °C) | 99th Percentile (26.5 °C) Relative to 90th Percentile (25.4 °C) | 1st Percentile (11 mm) Relative to 10th Percentile (24 mm) | 99th Percentile (302 mm) Relative to 90th Percentile (215 mm) | 1st Percentile (1.7 kn) Relative to 10th Percentile (2.7 kn) | 99th Percentile (5.7 kn) Relative to 90th Percentile (4.1 kn) |
| 40 | −2.97(−4.12, −1.80) | 4.68(3.17,6.21) | 0.05(−0.15,0.25) | 4.48(3.24,5.73) | 3.63(2.59,4.68) | 0.10(−2.54,2.81) |
| 41 | −2.96(−4.11, −1.79) | 4.75(3.24,6.28) | 0.05(−0.15,0.26) | 4.44(3.21,5.68) | 3.67(2.63,4.72) | 0.07(−2.57,2.77) |
| 42 | −2.94(−4.09, −1.78) | 4.80(3.30,6.33) | 0.06(−0.14,0.26) | 4.39(3.17,5.64) | 3.71(2.67,4.76) | 0.03(−2.59,2.73) |
| 43 | −2.93(−4.07, −1.77) | 4.85(3.35,6.38) | 0.07(−0.13,0.27) | 4.35(3.13,5.58) | 3.75(2.71,4.79) | 0.00(−2.62,2.68) |
| 44 | −2.92(−4.06, −1.76) | 4.90(3.40,6.42) | 0.07(−0.12,0.27) | 4.30(3.08,5.53) | 3.78(2.75,4.83) | −0.04(−2.64,2.64) |
| 45 | −2.90(−4.03, −1.75) | 4.94(3.44,6.45) | 0.08(−0.12,0.28) | 4.25(3.04,5.47) | 3.81(2.78,4.86) | −0.07(−2.66,2.59) |
| 46 | −2.88(−4.01, −1.74) | 4.97(3.48,6.48) | 0.09(−0.11,0.28) | 4.20(2.99,5.41) | 3.84(2.81,4.89) | −0.10(−2.68,2.55) |
| 47 | −2.86(−3.99, −1.73) | 4.99(3.51,6.50) | 0.09(−0.10,0.29) | 4.14(2.95,5.35) | 3.87(2.84,4.91) | −0.13(−2.70,2.50) |
| 48 | −2.84(−3.96, −1.72) | 5.01(3.54,6.51) | 0.10(−0.10,0.30) | 4.08(2.90,5.28) | 3.90(2.87,4.93) | −0.16(−2.71,2.46) |
| 49 | −2.82(−3.93, −1.70) | 5.03(3.56,6.52) | 0.11(−0.09,0.30) | 4.02(2.84,5.21) | 3.92(2.89,4.95) | −0.19(−2.72,2.41) |
| 50 | −2.80(−3.90, −1.69) | 5.03(3.57,6.52) | 0.11(−0.08,0.31) | 3.96(2.79,5.14) | 3.94(2.91,4.97) | −0.22(−2.73,2.36) |
| 51 | −2.78(−3.87, −1.68) | 5.04(3.58,6.51) | 0.12(−0.07,0.31) | 3.90(2.73,5.07) | 3.95(2.93,4.99) | −0.24(−2.74,2.32) |
| 52 | −2.75(−3.83, −1.66) | 5.03(3.59,6.50) | 0.13(−0.07,0.32) | 3.83(2.68,5.00) | 3.97(2.90,4,50) | −0.27(−2.74,2.27) |
| 53 | −2.73(−3.80, −1.65) | 5.02(3.59,6.48) | 0.13(−0.06,0.32) | 3.76(2.62,4.92) | 3.98(2.96,5.02) | −0.29(−2.75,2.23) |
| 54 | −2.70(−3.76, −1.63) | 5.01(3.58,6.45) | 0.14(−0.05,0.33) | 3.69(2.56,4.84) | 3.99(2.97,5.03) | −0.32(−2.75,2.18) |
| 55 | −2.68(−3.73, −1.61) | 4.99(3.57,6.42) | 0.15(−0.04,0.33) | 3.62(2.50,4.76) | 4.00(2.98,5.04) | −0.34(−2.75,2.14) |
| 56 | −2.65(−3.69, −1.60) | 4.96(3.56,6.39) | 0.15(−0.03,0.34) | 3.55(2.43,4.67) | 4.01(2.98,5.04) | −0.36(−2.75,2.09) |
| 57 | −2.62(−3.65, −1.58) | 4.93(3.54,6.35) | 0.16(−0.03,0.35) | 3.47(2.37,4.59) | 4.01(2.99,5.05) | −0.38(−2.75,2.05) |
| 58 | −2.59(−3.61, −1.56) | 4.90(3.51,6.31) | 0.17(−0.02,0.35) | 3.39(2.30,4.50) | 4.01(2.99,5.05) | −0.40(−2.75,2.00) |
| 59 | −2.56(−3.56, −1.54) | 4.86(3.48,6.26) | 0.17(−0.01,0.36) | 3.31(2.23,4.41) | 4.02(2.99,5.06) | −0.42(−2.75,1.96) |
| 60 | −2.53(−3.52, −1.52) | 4.82(3.45,6.21) | 0.18(0.00,0.36) | 3.23(2.16,4.32) | 4.01(2.98,5.06) | −0.44(−2.75,1.92) |

**Table S4.** *Cont.*

| Lag (Days) | Overall Percentage Change (95% CI) | | | | | |
|---|---|---|---|---|---|---|
| | Minimum Temperature (°C) | | Cumulated Rainfall (mm) | | Wind Speed (knots) | |
| | 1st Percentile (22 °C) Relative to 10th Percentile (23 °C) | 99th Percentile (26.5 °C) Relative to 90th Percentile (25.4 °C) | 1st Percentile (11 mm) Relative to 10th Percentile (24 mm) | 99th Percentile (302 mm) Relative to 90th Percentile (215 mm) | 1st Percentile (1.7 kn) Relative to 10th Percentile (2.7 kn) | 99th Percentile (5.7 kn) Relative to 90th Percentile (4.1 kn) |
| 61 | −2.49(−3.48, −1.50) | 4.77(3.41,6.16) | 0.19(0.00,0.37) | 3.15(2.08,4.23) | 4.01(2.97,5.06) | −0.46(−2.74,1.88) |
| 62 | −2.46(−3.43, −1.48) | 4.72(3.36,6.10) | 0.19(0.01,0.37) | 3.07(2.01,4.14) | 4.01(2.97,5.06) | −0.48(−2.74,1.84) |
| 63 | −2.42(−3.39, −1.45) | 4.67(3.31,6.04) | 0.20(0.02,0.38) | 2.98(1.93,4.05) | 4.00(2.95,5.06) | −0.49(−2.73,1.80) |
| 64 | −2.39(−3.34, −1.43) | 4.61(3.25,5.98) | 0.21(0.03,0.38) | 2.90(1.86,3.95) | 3.99(2.94,5.06) | −0.51(−2.73,1.76) |
| 65 | −2.35(−3.30, −1.40) | 4.54(3.19,5.91) | 0.21(0.03,0.39) | 2.81(1.78,3.86) | 3.98(2.92,5.05) | −0.53(−2.73,1.73) |
| 66 | −2.32(−3.25, −1.38) | 4.48(3.13,5.85) | 0.22(0.04,0.40) | 2.72(1.69,3.76) | 3.97(2.91,5.05) | −0.54(−2.72,1.69) |
| 67 | −2.28(−3.20, −1.35) | 4.41(3.06,5.78) | 0.23(0.05,0.40) | 2.63(1.61,3.66) | 3.96(2.88,5.05) | −0.56(−2.72,1.66) |
| 68 | −2.24(−3.16, −1.32) | 4.34(2.98,5.71) | 0.23(0.06,0.41) | 2.54(1.53,3.57) | 3.95(2.86,5.05) | −0.57(−2.72,1.63) |
| 69 | −2.20(−3.11, −1.29) | 4.26(2.90,5.64) | 0.24(0.06,0.42) | 2.45(1.44,3.47) | 3.93(2.83,5.04) | −0.58(−2.72,1.60) |
| 70 | −2.17(−3.07, −1.26) | 4.18(2.82,5.56) | 0.25(0.07,0.42) | 2.36(1.35,3.37) | 3.92(2.81,5.04) | −0.60(−2.72,1.58) |
| 71 | −2.13(−3.02, −1.22) | 4.10(2.73,5.49) | 0.25(0.08,0.43) | 2.26(1.26,3.27) | 3.90(2.78,5.03) | −0.61(−2.72,1.55) |
| 72 | −2.09(−2.98, −1.19) | 4.02(2.64,5.42) | 0.26(0.08,0.44) | 2.17(1.17,3.17) | 3.88(2.74,5.03) | −0.62(−2.73,1.53) |
| 73 | −2.05(−2.93, −1.15) | 3.93(2.54,5.34) | 0.27(0.09,0.44) | 2.07(1.07,3.08) | 3.86(2.71,5.03) | −0.63(−2.73,1.51) |
| 74 | −2.00(−2.89, −1.11) | 3.84(2.44,5.27) | 0.27(0.10,0.45) | 1.97(0.98,2.98) | 3.84(2.67,5.02) | −0.64(−2.74,1.50) |
| 75 | −1.96(−2.85, −1.07) | 3.75(2.33,5.19) | 0.28(0.10,0.46) | 1.88(0.88,2.88) | 3.82(2.64,5.02) | −0.65(−2.75,1.49) |
| 76 | −1.92(−2.81, −1.03) | 3.66(2.22,5.12) | 0.29(0.11,0.47) | 1.78(0.78,2.78) | 3.80(2.59,5.02) | −0.67(−2.76,1.48) |
| 77 | −1.88(−2.77, −0.98) | 3.56(2.11,5.04) | 0.29(0.11,0.47) | 1.68(0.68,2.69) | 3.78(2.55,5.01) | −0.68(−2.78,1.47) |
| 78 | −1.84(−2.73, −0.94) | 3.47(1.99,4.97) | 0.30(0.12,0.48) | 1.58(0.58,2.59) | 3.75(2.51,5.01) | −0.69(−2.79,1.47) |
| 79 | −1.79(−2.69, −0.89) | 3.37(1.87,4.90) | 0.31(0.12,0.49) | 1.48(0.48,2.50) | 3.73(2.46,5.01) | −0.70(−2.81,1.46) |
| 80 | −1.75(−2.65, −0.84) | 3.27(1.74,4.82) | 0.31(0.13,0.50) | 1.38(0.37,2.40) | 3.70(2.42,5.00) | −0.70(−2.83,1.47) |
| 81 | −1.71(−2.61, −0.79) | 3.17(1.62,4.75) | 0.32(0.13,0.51) | 1.28(0.26,2.31) | 3.68(2.37,5.00) | −0.71(−2.85,1.47) |

**Table S4.** *Cont.*

| Lag (Days) | Overall Percentage Change (95% CI) | | | | | |
|---|---|---|---|---|---|---|
| | Minimum Temperature (°C) | | Cumulated Rainfall (mm) | | Wind Speed (knots) | |
| | 1st Percentile (22 °C) Relative to 10th Percentile (23 °C) | 99th Percentile (26.5 °C) Relative to 90th Percentile (25.4 °C) | 1st Percentile (11 mm) Relative to 10th Percentile (24 mm) | 99th Percentile (302 mm) Relative to 90th Percentile (215 mm) | 1st Percentile (1.7 kn) Relative to 10th Percentile (2.7 kn) | 99th Percentile (5.7 kn) Relative to 90th Percentile (4.1 kn) |
| 82 | −1.66(−2.58, −0.73) | 3.07(1.48,4.68) | 0.33(0.14,0.52) | 1.18(0.16,2.21) | 3.65(2.32,5.00) | −0.72(−2.88,1.48) |
| 83 | −1.62(−2.55, −0.68) | 2.97(1.35,4.60) | 0.34(0.14,0.53) | 1.08(0.05,2.12) | 3.62(2.27,5.00) | −0.73(−2.91,1.49) |
| 84 | −1.57(−2.51, −0.62) | 2.86(1.22,4.53) | 0.34(0.15,0.54) | 0.98(−0.06,2.03) | 3.60(2.22,5.00) | −0.74(−2.94,1.51) |
| 85 | −1.53(−2.48, −0.56) | 2.76(1.08,4.46) | 0.35(0.15,0.55) | 0.87(−0.18,1.94) | 3.57(2.16,5.00) | −0.75(−2.97,1.52) |
| 86 | −1.48(−2.45, −0.50) | 2.65(0.94,4.39) | 0.36(0.16,0.56) | 0.77(−0.29,1.85) | 3.54(2.11,4.99) | −0.76(−3.01,1.54) |
| 87 | −1.44(−2.42, −0.44) | 2.55(0.80,4.32) | 0.36(0.16,0.57) | 0.67(−0.40,1.76) | 3.51(2.05,4.99) | −0.77(−3.05,1.57) |
| 88 | −1.39(−2.40, −0.38) | 2.44(0.66,4.26) | 0.37(0.16,0.58) | 0.57(−0.52,1.67) | 3.49(2.00,5.00) | −0.77(−3.09,1.59) |
| 89 | −1.35(−2.37, −0.32) | 2.33(0.51,4.19) | 0.38(0.17,0.59) | 0.47(−0.63,1.58) | 3.46(1.94,5.00) | −0.78(−3.13,1.62) |
| 90 | −1.30(−2.34, −0.25) | 2.23(0.37,4.12) | 0.38(0.17,0.60) | 0.36(−0.75,1.49) | 3.43(1.89,5.00) | −0.79(−3.17,1.65) |

**Figure S1.** Autocorrelation and partial autocorrelations of residuals for the selected model.

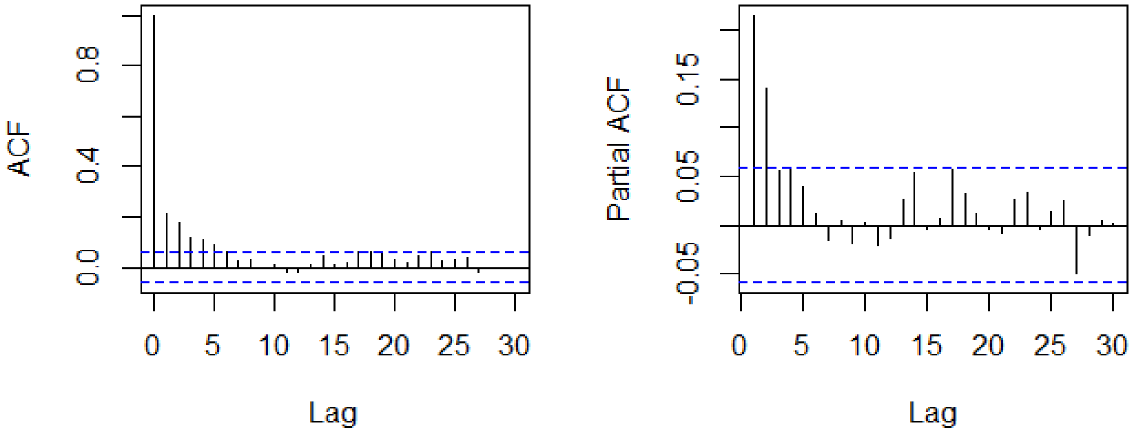


## References

1. R Development Core Team. R: A Language and Environment for Statistical Computing; R Foundation for Statistical Computing: Vienna, Austria, 2013. Available online: http://www.r-project.org/ (accessed on 15 April 2013).
2. Adler, D.; Murdoch, D. 3D Visualization Device System (OpenGL). 2012. Available online: http://rgl.neoscientists.org/about.shtml (accessed on 17 October 2013).
3. Burnham, K.P.; Anderson, D.R. Multimodel inference—Understanding AIC and BIC in model selection. *Sociol. Method. Res.* **2004**, *33*, 261–304.

© 2013 by the authors; licensee MDPI, Basel, Switzerland. This article is an open access article distributed under the terms and conditions of the Creative Commons Attribution license (http://creativecommons.org/licenses/by/3.0/).
