# Supplementary figures and images for: Assessing Weather Effects on Dengue Disease in Malaysia
Source: Int J Environ Res Public Health. 2013 Nov 26;10(12):6319–34. doi: 10.3390/ijerph10126319 (PMC3881116; doi:10.3390/ijerph10126319)

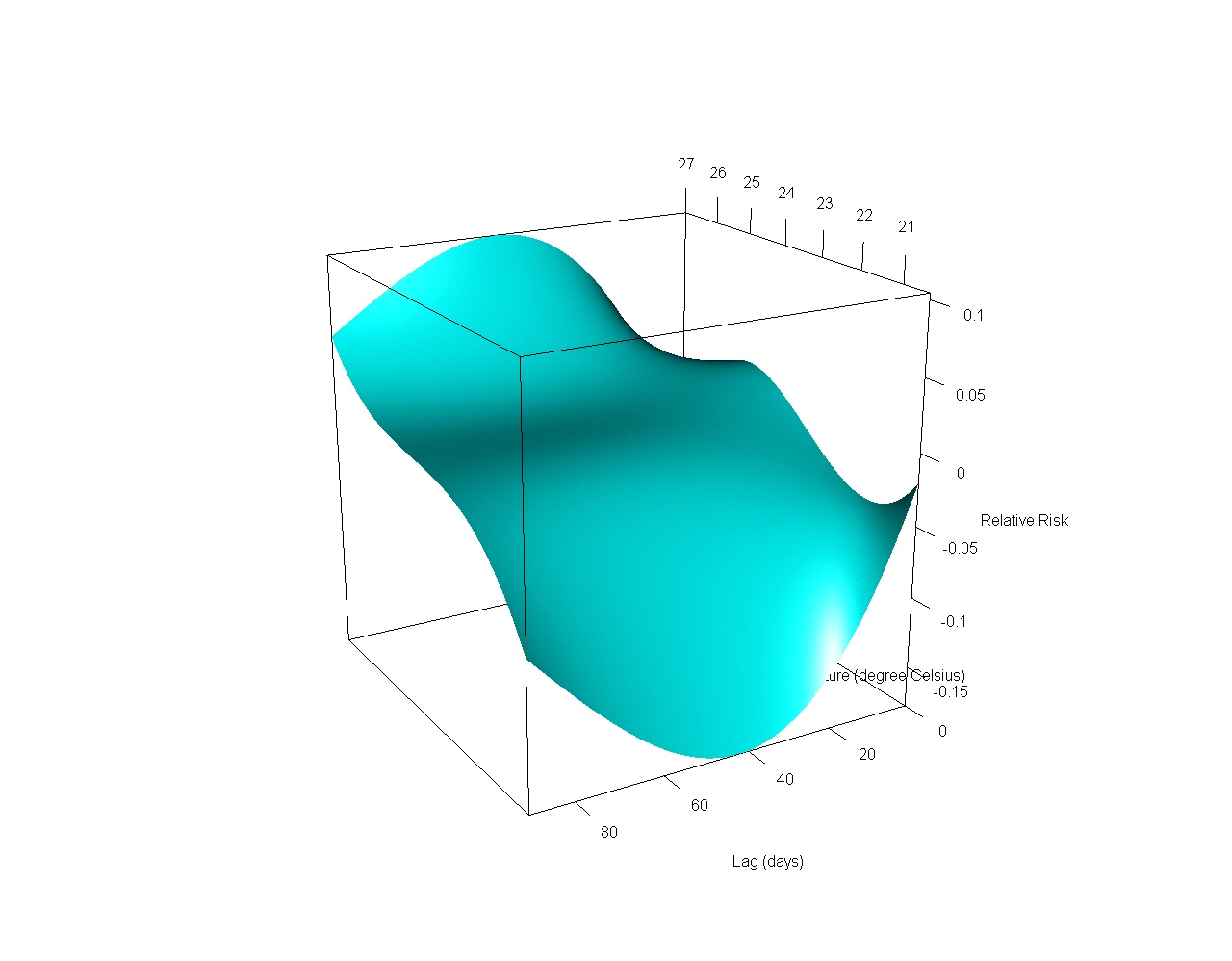

Supplement: Supplementary File 2 — (GIF, 4560 KB) [file ijerph-10-06319-s002.gif]

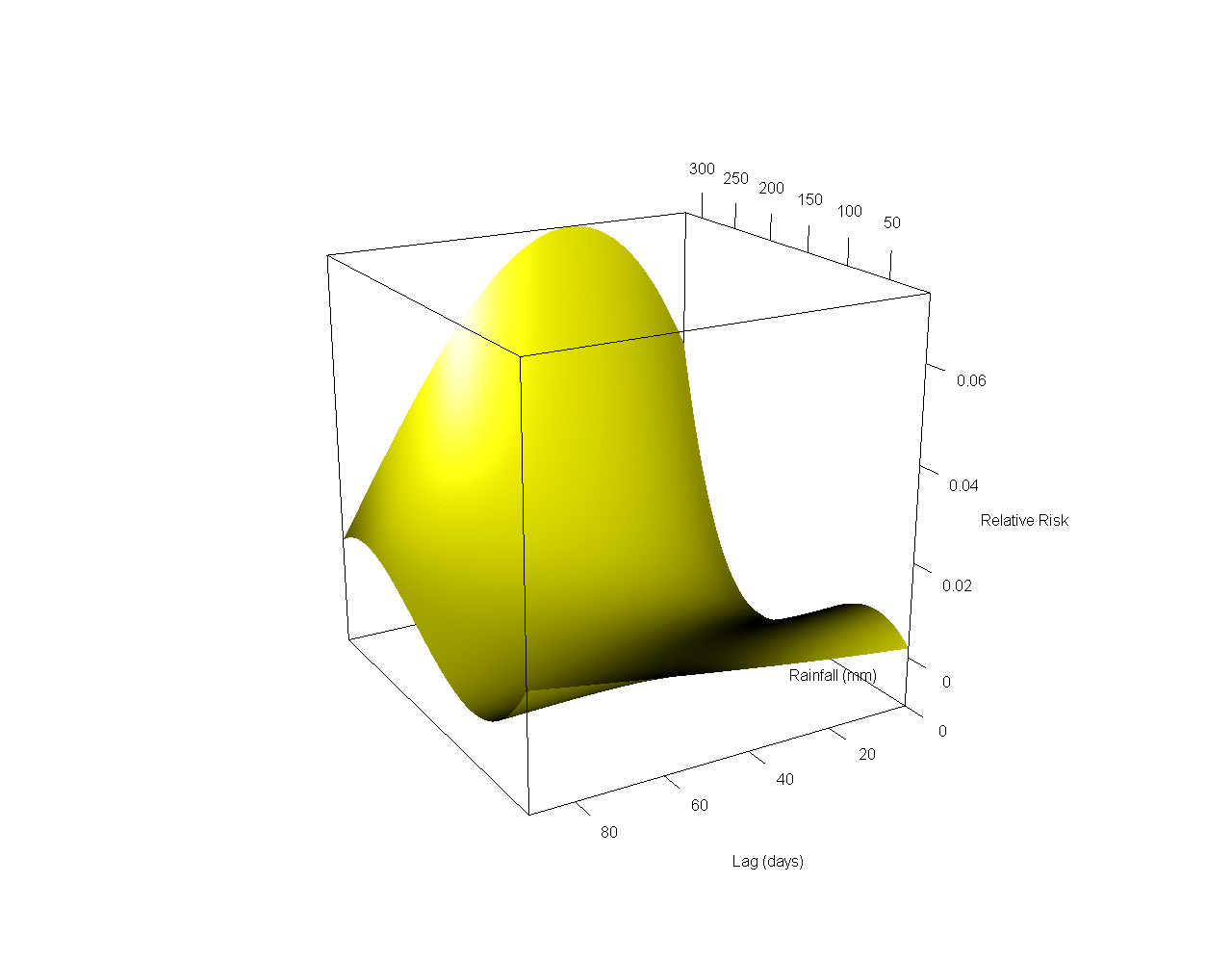

Supplement: Supplementary File 3 — (GIF, 5039 KB) [file ijerph-10-06319-s003.gif]

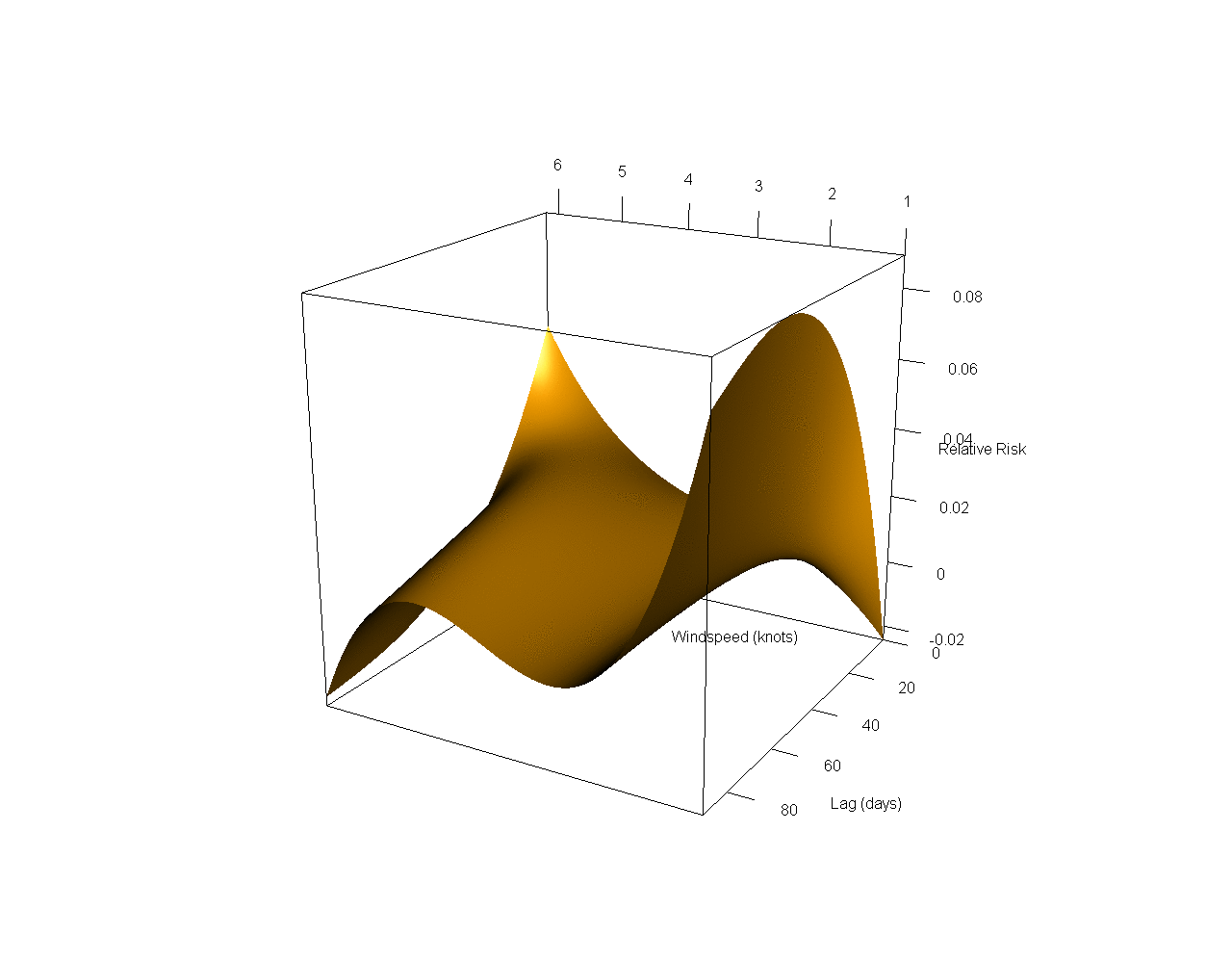

Supplement: Supplementary File 4 — (GIF, 5995 KB) [file ijerph-10-06319-s004.gif]
